# ICSI diagnostic: a way to prevent total fertilization failure after 4 unsuccessful IUI

**DOI:** 10.1186/s12610-017-0061-z

**Published:** 2017-09-21

**Authors:** Arnaud Larbuisson, Dominique Raick, Stephanie Demelenne, Annick Delvigne

**Affiliations:** 1 A.R.T. Laboratory of C.H.C. St Vincent, 4000 Rocourt, Belgium; 2 A.R.T medical Team of C.H.C. St Vincent, 4000 Rocourt, Belgium

**Keywords:** Fertilization rate - Intracytoplasmic sperm injection, In vitro fertilization - Pregnancy, Total fertilization failure, Taux de fertilisation, Injection de sperme intra cytoplasmique, Fécondation in vitro, Grossesse, Échec total de fécondation

## Abstract

**Background:**

The aim of this retrospective study is to investigate the relevance of dividing oocytes and using some for traditional in vitro fertilization (IVF) and others for intracytoplasmic sperm injection (ICSI) as of the first IVF cycle in patients with unexplained infertility who have undergone 4 intrauterine insemination (IUI) cycles which produced no pregnancies.

**Methods:**

This retrospective study includes patients with unexplained infertility who have failed to become pregnant, after 4 IUI, despite normal semen parameters after sperm capacitation. These women were treated in our assisted fertilization program from 2008 until 2015. We analysed the first cycles of women in whom more than 4 oocyte cumulus complexes (OCC) were retrieved and single embryo transfer was performed.

**Results:**

Dividing oocytes between two fertilization techniques reduce the rate of total fertilization failure during the first IVF cycle. No statistical difference were observed for 2 pronuclei (PN) rate between the two techniques. On the other hand, we observed a significantly lower rate of 3 PN, 1 PN, 0 PN with ICSI in comparison with conventional fertilization.

**Conclusions:**

Splitting the oocytes between classical IVF and ICSI increases the chance of embryo transfer on a first IVF cycle after 4 unsuccessful IUI cycles. This half-and-half policy reduces the risk, for the infertile couple, of facing total failure of fertilization and also can provide useful information for the next attempts.

## Résumé

**Contexte:**

L’objectif de cette étude rétrospective est. de montrer la pertinence de répartir les ovocytes ponctionnés entre deux méthodes de fécondation, la fécondation classique (IVF) et l’injection de sperme intra cytoplamisque (ICSI), lors d’un premier cycle de stimulation après 4 essais insémination intra-utérine (IUI) infructueuses.

**Méthodes:**

Cette étude rétrospective inclut les patientes ayant réalisés 4.

inséminations intra utérine sans grossesse entre 2008 et 2015. Les paramètres du sperme sont normaux. Nous avons analysé les cycles de stimulation de rang 1 des patientes où minimum 4 ovocytes ont été prélevés et un seul embryon transféré.

**Résultats:**

Répartir les ovocytes entre les deux techniques de fécondation réduit le taux d’échec de fécondation total lors du premier cycle de stimulation. Aucune différence statistique n’a été observée entre les taux de 2 pronuclei (PN) pour les deux méthodes de fécondation. Toutefois une réduction significative est. observée pour les taux de 3 PN, 1 PN et 0 PN en faveur de l’ICSI.

**Conclusion:**

La répartition des ovocytes lors d’un premier essai de fécondation in vitro réalisé après 4 échecs d’insémination intra-utérine, permet d’augmenter les chances d’aboutir à un transfert d’embryons. Cette procédure réduit les risques d’avoir un échec total de fécondation chez des couples infertile et permet d’obtenir des informations pour les essais ultérieurs.

## Introduction

About 30% of all couples experience unexplained infertility [1]. Intrauterine insemination (IUI) should be the first Assisted Reproductive Technology (ART) used in such cases. The absence of pregnancy after IUI can be related to many difficult to diagnose factors such as poor oocyte quality, sperm dysfunction, fertilization defect, embryo implantation failure… In vitro fertilization should then be the next step in the ART process. Different studies have shown that classical fertilization (IVF) leads to total failure of fertilization in 5–25% [2–7] of couples with unexplained infertility. Although intracytoplasmic sperm injection (ICSI) is a widely accepted technique for confirmed male infertility, it is not consistently used for the first IVF cycle in couples with unexplained infertility. Several studies do show, however, that total fertilization failure could be avoided by using one or the other of the two fertilization techniques, on half of the collected oocytes, respectively.

The aim of this retrospective study is to investigate the relevance of dividing oocytes and using some for traditional IVF and others for ICSI as of the first IVF cycle in patients with unexplained infertility who have undergone 4 IUI cycles which produced no pregnancies.

## Methods

### Study design

This retrospective study includes patients with unexplained infertility who have failed to become pregnant, after 4 IUI, despite normal semen parameters of World Health Organisation (WHO) criteria [8] after sperm capacitation. These women were treated in our assisted fertilization program from 2008 until 2015. We included women between 18 and 43 years of age. We began by analysing only the first cycles of women in whom more than 4 oocyte cumulus complexes (OCC) were retrieved, after stimulation. Sibling oocytes were randomly divided between the two fertilization methods: classical IVF (IVF) and ICSI. We observed cycles in which a single embryo transfer was performed and excluded all others.

We then analysed the second stimulation cycle in a small cohort of similar patients who had been treated with complete classical fertilization technique according to the result of the first trial.

### Sperm preparation

On the day of oocyte retrieval, sperm analysis was performed according to the WHO laboratory standards of human semen and sperm. Motile spermatozoa were selected using a Sil Select two layer gradient centrifugation (FertiPro, Beernem, Belgium) for 15 min followed by two 5 min cleansings in G-IVF (Vitrolife, Goteborg, Sweden).

Our sperm criteria required in order to perform the 4 first IUIs were the obtaining of at least 1 million motile spermatozoa, at least 4% of normal sperm and ≥32% progressive motility based on strict sperm morphology criteria (WHO), in the final sperm preparation aliquot (500 μl).

Our criteria for the final sperm preparation to perform classical IVF fertilization are 1–1, 5.10^5^ motile spermatozoa per millilitre and >4% morphologically normal spermatozoa. If the final preparation does not meet these criteria during the first IVF cycle, ICSI is performed and this couple is excluded from the study.

### Stimulation protocol

During IVF trials, stimulation is achieved with 100 to 300 IU of gonadotrophins depending on age, body mass index (BMI) and the Anti-Mullerian hormone (AMH) level (FSHrec: Puregon,; MSD, Oss, the Netherlands; GonalF: Merck-Serono, Geneva, Switzerland) or human Menopausal Gonadotrophin (Menopur, Ferring, Alost, Belgique). Ovulation was controlled by either gonadotropin-releasing hormone (GnRH) antagonist (Cetrorelix, Merck Serono or Ganirelix, MSD) or GnRH agonist (Buserelin: Suprefact; Sanofi-Avantis, Diegem, Belgium or triptoréline: Gonapeptyl, Ferring) respectively in 68% (170 cycles) and 32% (81 cycles) of cases. The GnRH antagonist is administered according to a flexible protocol which is once daily, when at least one follicle has reached a diameter of 14 mm and / or the oestradiol (E2) level is at 400 pg / ml. When a GnHR agonist is used, the long protocol is applied in the majority of such cycles. Triggering was obtained with 5000 IU of human chorionic gonadotrophin (hCG) (Pregnyl, MSD, Bruxelles, Belgium).

### Oocytes fertilization

For conventional fertilization, OCC were inseminated over-night with a sperm suspension at a concentration of 100–150.10^3^ motile spermatozoa/ml. Oocytes selected for ICSI fertilization were stripped of their cumulus using Hyase (Vitrolife, Goteborg, Sweden) 40 IU/ml. Sperm injection was performed only on metaphase II oocytes, four hours after pick up.

### Fertilization check, embryo culture and transfer

Between 16 and 18 h after injection or classical insemination, normal fertilization was confirmed by the presence of two pronuclei (PN). Abnormal fertilization was determined when 0, 1 and 3 PN were obtained. We excluded immature oocytes (0 polar body (PB) and germinal vesicle (GV)) from the count of conventional fertilization after removal of cumulus cells on day 1. The fertilization rate was expressed per mature oocytes injected or inseminated. Total fertilization failure (TFF) is defined as the absence of two pronuclei (2PN) embryos on day 1 after insemination. Embryos were cultured in an incubator at 37 °C, under 5% of CO_2_ and 6% of O_2_ and were assessed each morning until their transfer (day 3 or day 5) based on Istanbul consensus criteria [9]. The embryo with the best morphological score was selected for transfer and only one embryo was transferred (SET). Supernumerary embryos of good quality were vitrified. The fertilization method is not a criterion for embryo selection in this study. When more than one embryo obtained the same morphological score, we analysed their kinetic development and selected the one which had the best one [10–14].

### Pregnancy

An hCG blood test was performed 15 days after the oocytes pick-up (OPU) and then 7 days after that. A developing pregnancy was confirmed by observation of an intrauterine gestational sac and foetal heart beat 10 days after the second blood test.

### Statistical analysis

The analysis of the results was carried out using Fisher’s test and the Chi-square test when it was appropriate. Statistical significance was accepted when *p-value* < 0, 05. The analysis was performed on GraphPad^©^ Prims v5**.**

## Results

### Same fertilization rate between conventional fertilization method and ICSI

A total of 251 IVF cycles were performed using both fertilization methods after 4 unsuccessful IUI cycles. The mean age of the women was 30, 7 years. An average of 6 OCC/cycle were obtained by pick up. Classical IVF and ICSI outcomes from sibling oocytes are shown in Table 1. The fertilization rate (2 PN) with ICSI was slightly higher than with IVF but had no statistical significance (69% vs 67, 5%; *p-value > 0, 5*). On the other hand, we observed a significantly lower rate of 3 PN, 1 PN, 0 PN with ICSI compared to IVF. In 22 cycles, a total failure of fertilization was observed with the IVF method whereas no fertilization failure occurred with ICSI for sibling oocytes.

**Table 1:** Comparison of fertilization rates between IVF and ICSI technics on the first cycle after 4 IUI

	IVF	ICSI	*p*-value
Number of cycles	251		
MII oocytes	749	679	
2 PN (%)	506 (67, 5%)	466 (69%)	NS
3 PN (%)	52 (7%)	19 (3%)	0,0003
1 PN (%)	42 (6%)	19 (3%)	0,0087
0 PN (%)	161 (21%)	106 (16%)	0,0044
Total failure of fertilization (%)	22 (9%)	0	<0.0001

*IVF* in vitro fertilization, *ICSI* Intracytoplamic sperm injection, *MII* metaphase 2, *NS* no significant, *PN* pronuclei

Of the 22 couples who experienced total fertilization failure, 7 came back for a second attempt. ICSI was performed on the entire oocyte cohort (Table 2). No total fertilization failure occurred on this second cycle and a normal fertilization rate was observed (82%).

**Table 2:** Fertilization rates between IVF and ICSI technics on the second cycle

	IVF_T2	ICSI_T2
No of cycles	23	7
No of OCC /MII	168	51
2 PN (%)	100 (60%)	42 (82%)
3 PN (%)	7 (4%)	2 (4%)
1 PN (%)	(11%)	0
0 PN (%)	32 (19%)	4 (8%)
Total failure of fertilization (%)	0	0

*IVF_T2* in vitro fertilization on second cycle, *ICSI_T2* Intracytoplamic sperm injection on second cycle, *OCC* Oocyte cumulus complex, *MII* metaphase 2, *NS* no significant, *PN* pronuclei

Regarding cycles for which fertilization was obtained by classical IVF on the first attempt, 23 couples came back for a second trial and classical IVF was performed on the whole cohort with a fertilization rate of 60%. Total fertilization failure was never observed.

### Pregnancy evolution regarding IVF or ICSI

Results of pregnancy outcome after single embryo transfer (SET) during the first cycle are shown in Table 3. Out of 251 cycles, 136 SETs were performed with embryos obtained using the IVF method as were 115 SETs with embryos from ICSI. No statistical difference was observed between the two fertilization methods for hCG+ level per SET (57% vs 60%), ongoing pregnancy rates (40% vs 50%); biochemical pregnancies (10% vs 4%); or number of miscarriages (10% vs 5%). The cryopreservation rate of embryos is the same for the two technics (28%).

**Table 3:** Distribution of pregnancy between IVF and ICSI technics on the first attempt

	IVF	ICSI	*p*-value
No of transfers	136	115	
hCG + per ET (%)	77 (57%)	69 (60%)	NS
Ongoing pregnancy per ET (%)	54 (40%)	57 (50%)	NS
Biochemical pregnancy (%)	13 (10%)	4 (3%)	NS
Miscarriage per ET (%)	8 (10%)	6 (5%)	NS
Cryopreservation rate/OCC	28%	28%	NS

*ET* Embryo transfer, *IVF* in vitro fertilization, *ICSI* Intracytoplamic sperm injection, *OCC* Oocyte cumulus complex, *NS* no significant

## Discussion

In Belgium, the social security services will contribute financially to up to 6 IVF cycles of treatment (including IVF cycle reaching the fertilisation step with or without transfer) with the hope that patients will be able to constitute their entire family. Under these conditions, the loss of even one chance of embryo transfer has financial and psychological repercussions for patients and must be avoided. Our study has shown that after 4 unsuccessful IUI attempts, 9% of the first IVF cycles led to total fertilization failure when conventional fertilization was performed. On the sibling oocytes treated by ICSI, no total fertilization failures occurred. Another retrospective study of 154 cycles has also shown the importance of splitting oocytes for both types of fertilization technique at the first cycle to avoid total fertilization failure (TFF). These authors obtained 7, 2% of TFF which was less than our 9%. This difference can be explained by the higher number of oocytes retrieved (17, 3 vs 6) per patient which may decrease oocyte quality [7]. Similarly, a meta-analysis published by Johnson et al. [6] considered 11 studies in which oocytes were divided between IVF and ICSI fertilization methods. Nine of these 11 reported TFF of IVF (6–43%) and 2/11 also reported TFF with ICSI and IVF. Our rate of TFF (9%) is in the range of this meta-analysis. The strength of our study in comparison to the meta-analysis is that we included all consecutive first cycles and only 1 cycle per couple. This protocol can explain some differences with the meta-analysis’ studies in which 1 cycle per couple is included but no information regarding which attempt it is appears. Although we conducted a retrospective analysis, our results concerned data from many more cycles than in the meta-analysis (251 vs 6–248).

Cycle who attained fertilization (through FIV and ICSI) presented no significant differences in the characteristics of the fertilized egg nor in the global fertilization rate, regardless of which technique had been used although total fertilization failure occurred in 9% of patient.The comparison of our hCG+ and ongoing pregnancy rates with SET showed better results with ICSI but the difference was not statistically significant.

Our results are comparable to those published in other studies that show that in cases of unexplained sterility, fertilization techniques did not influence embryo quality nor the pregnancy rate [3, 5, 7, 15].

The policy of the double fertilization technique in cases of idiopathic infertility with IUI failure prevents the cancellation of embryo transfer on the first IVF attempt due to a total failure of fertilization. Furthermore, this protocol provides not only treatment but also diagnoses. It allows us to analyze the fertilizing capacity of sperm which cannot be assessed by conventional sperm analysis. Thanks to this diagnostic technique, we can attribute unexplained infertility to the male and treat accordingly for the next attempts.

Based on the fertilization rate (FR) of the first cycle, we decide which fertilization method should be used for the next cycle and not only in cases of total fertilization failure. Indeed, in cases of fertilization failure or a low fertilization rate (<50%), with conventional fertilization at the first cycle, we made the decision to perform ICSI on all of the oocytes for next one. Our small cohort of second cycles confirms the merit of this procedure since no total fertilization failures occurred. However, due to the small number of cases, more second cycles must be studied in order to confirm these preliminary observations and validate this protocol. These results should also take into account previous studies which showed that the rate of total fertilization failure during second attempts decreased after total fertilization failure during the first one [16, 17] in which classical fertilization was used Barlow et al. and Ben-Shlomo et al. assert that when normal or mildly defective sperm is used, fertilization failure (FF) at the first cycle has no predictive value regarding the next one. However, the small size of the cohorts in these studies limits the strength of their conclusions.

In conclusion, splitting the oocytes between classical IVF and ICSI increases the chance of embryo transfer on a first IVF cycle after 4 unsuccessful IUI cycles. This half-and-half policy reduces the risk, for the infertile couple, of facing total failure of fertilization and also can provide useful information for the next attempts. Nevertheless, the adequacy of this attitude should be confirmed by the analysis of a larger cohort of second cycle in which total fertilization failures would be confirmed.

## Abbreviations

AMH: Anti-Mullerian Hormone
BMI: Body Mass Index
E2: Oestradiol
ET: Embryo transfer
FF: Fertilization failure
FR: Fertilization rate
GnRH: Gonadotropin Releasing Hormone
GV: Germinal vesicle
hCG: Human Chorionic Gonadotrophin
ICSI: Intracytoplasmic sperm injection
IUI: Intra Uterin Insemination
IVF: In vitro fertilization
OCC: Oocyte Cumulus Complexes
OPU: Oocytes Pick Up
PB: Polar body
PN: Pronuclei
SET: Single embryo transfer
TFF: Total fertilization failure
